# IMPACT OF GRANDPARENTS’ CHRONIC STRESS ON GRANDPARENT-PARENT RELATIONSHIP AND LIFE SATISFACTION BY GENDER AND RACE

**DOI:** 10.1093/geroni/igad104.3413

**Published:** 2023-12-21

**Authors:** Nusrat E Mozid, Kate Guastaferro

**Affiliations:** New York University, New York City, New York, United States; New York University, New York City, New York, United States

## Abstract

Grandparents raising grandchildren are a rapidly growing population in the United States—an estimated 7.1 million children under 18 live with a grandparent in 2023. Family system theory suggests raising a grandchild impacts grandparents’ immediate and extended family relationships and amplifies stressors in the relationship with the parent of the grandchild. The quality of relationships with the parents negatively impacts grandparents’ psychosocial well-being but may also have lasting adverse effects on the grandchild. Using the most recent wave (2020) of the nationally representative Health and Retirement Study, we conducted linear regression analyses to understand the association between grandparents raising grandchildren’s (N=4,238) chronic stress (e.g., health, finances, housing) and anxiety symptoms by gender and race. Then, we examined the grandparents’ relationship with the parent and satisfaction with life as potential mediators in these associations. Results suggest grandparents’ stressors were significantly associated with anxiety symptoms (β=0.349; p< 0.01); however, there were no significant differences in total chronic stress by either gender or race. Path analysis showed both the grandparent-parent relationship and grandparents’ satisfaction with life partially mediated the association between grandparents’ chronic stress and anxiety symptoms (p< 0.001). Cultivating a consistent and quality relationship between grandparent and parent as well as focusing on grandparents’ perspectives of life suggests innovative intervention targets to improve the health and well-being of both grandparents and grandchildren.

